# TVET as an important factor in country’s economic development

**DOI:** 10.1186/2193-1801-3-S1-K3

**Published:** 2014-12-04

**Authors:** Margarita Pavlova

**Affiliations:** UNESCO-UNEVOC Centre (Hong Kong), Hong Kong Institute of Education, Kragujevac, Hong Kong

Education and training for productive employment is vital for economic and social development in Asia and the Pacific. Technical and Vocational Education and Training (TVET) is viewed as a tool for productivity enhancement and poverty reduction in the region. As there is a strong correlation between the proportion of TVET students at the post-secondary level (tertiary, non-degree, ISCED 5b) and per capita income [1] many countries have taken steps to strengthen policy guidance and regulatory frameworks for technical and vocational education and training and to improve partnerships with private sector and employers. However, there is a difference between developed and less developed countries in terms of their first priorities regarding TVET. The first group of countries emphasize quality improvement, monitoring and evaluation of TVET, the availability of national development plans, but the second group of countries focus on the cost of enrolment and implementation of TVET [2]. Although skilled human resources are the primary asset of many countries in the region, an inadequately educated workforce is still among the most problematic factors for doing business in many countries in Asia and the Pacific [3].

This presentation brings together a number of areas of strategic development for Hong Kong stated in the 2014 Chief Executive’s Policy Address; namely economic development, innovation and technology industries, vocational education and environmental protection. High performance in increasingly competitive global economies combined with the need to address global challenges posed by climate change and carbon emissions, environmental degradation and pollution, health, and poverty, require successful countries to adapt innovation-driven strategies for growth that should be supported by TVET. The importance of transitions towards greener economies has been recognized globally, by both regions and separate countries (e.g. The Europe 2020 Strategy [4]; The Central People's Government of the People's Republic of China, 2011 [5]). Economic and Social Survey of Asia and the Pacific (ESCAP) [6] concludes that government policies that are directed towards strengthening *social and environmental pillars of sustainable* development could serve as “the solution to improving the quality of growth in the region” ([6] p.62). For Asia and the Pacific, the development of the “green economy” that recognizes the “important interlinkages between the environmental resource base, economic systems and social development” [7, p.50] could lead to investment and capacity development in such areas as renewable energy resources, green manufacturing sectors, urbanization, food security, sustainable agricultural practices and biodiversity. China, for example, identified the aim of developing a circular economy as one direction of their current Fifth year plan [5], the economy, where ‘reduce, reuse and recycle’ at every stage of production and consumption are promoted. The Government of Hong Kong SAR has invested strategically in innovation and technology as drivers for economic growth and competitiveness as due to environmental challenges, “faster and broader innovation of new technology is critical for achieving a sustainable future, [particularly], “the development of green industries” [8].

Economic changes caused by green restructuring should be supported by human resource development that results in significant impact on skills. **This presentation examines how TVET can contribute to green economic restructuring in the region through developing effective approaches towards formulation of green skills** (technical skills, knowledge, values and attitudes necessary for worker for the transition to an economy with a reduced negative impact on the environment) [9]. **It also evaluates greening of TVET in several Asian countries and argues that green innovation supported by governments could play an important role in transition towards a greener economy that in turn results in inclusion of green skills into a competence-based TVET.**

The presentation is based on the results of several research projects that were focused on different aspects of identifying, developing and including green skills in TVET to support transition to greener economies. As research in developing Asian countries demonstrates, although inclusion of new green competencies both generic and specific into curriculum has been observed, it is not enough on its own. There is a need for a holistic framework for greening TVET that includes development of values and attitudes (Pavlova, in press). While industry is slow in re-orienting itself towards greening, government is playing a crucial role in (i) adjusting TVET towards the needs of economy (both current and future), individuals and societies, and (ii) supporting green innovation development and diffusion for stimulating market demand in green skills that is particularly important for curriculum development in a competence-based TVET.
